# Stability analysis of soft–hard-interbedded anti-inclined rock slope

**DOI:** 10.1038/s41598-023-28657-2

**Published:** 2023-01-30

**Authors:** Jian‑jun Guo, Zhen-wei Wu, Kai Liu

**Affiliations:** 1 National Engineering Research Center for Inland Waterway Regulation, Chongqing Jiaotong University, Chongqing, 400074 China; 2 Chongqing Jiaotong University, Chongqing, 400074 China

**Keywords:** Hydrogeology, Natural hazards

## Abstract

The instability of rock slope is still a very frequent geological disaster, which seriously affects people's life and production activities. Previous studies have mainly focused on deformation mechanism, prediction, and control of hard rock with single lithology, while there are limited studies on the theoretic computational method of the stability for soft–hard interbedded anti-inclined rock strata. In this study, a geomechanical model for the toppling failure of soft–hard-interbedded anti-inclined rock slope is established. The modes of failure for soft and hard rock strata are analyzed, the computational formula of the downward thrust for each anti-inclined rock stratum is derived, and the stability safety factor of each rock stratum is defined. A theoretical computational method for determining the potentially most dangerous failure surface of soft–hard-interbedded anti-inclined rock slope is proposed. By comparing with the existing research results, the theoretical solving method proposed in this study can well solve the location of the potentially most dangerous failure surface of soft–hard-interbedded anti-inclined rock slope. The potentially most dangerous failure surface of this kind of slope is approximately planar, and the angle between it and the normal plane of the rock strata is an acute angle within 30°. It provides theoretical support for the stability analysis of this kind of slope.

## Introduction

In the mountainous areas of Southwest China, natural slopes and artificial slopes are widely distributed and numerous. In the anti-inclined rock slopes, large-scale landslide with large deformation and serious damage are often formed, moreover, the number of the toppling deformation and instability failure cases accounts for a large proportion in all kinds of large landslide cases^1^. There are many forms of toppling deformation and instability in this kind of anti-inclined rock slopes, among which block bending-toppling is one of the main toppling forms, this kind of failure is often formed in the anti-inclined rock mass, such as sandstone and mudstone interbedding, chert and shale interbedding, thin-layer limestone^2^. In the red rock beds of Jurassic System in Sichuan basin, Three Gorges Reservoir area, Yunnan Province and Guizhou Province, there are a large number of soft–hard-interbedded rock slopes. Especially in the Three Gorges Reservoir area, due to the interbedded lithologic combination of sandstone and mudstone formed by Jurassic strata, there are many cases of block bending-toppling failure of soft–hard-interbedded anti-inclined rock slope, which seriously threatens the safety of local people's lives and property, even restricts the local social and economic development. For example, Gongjiafang landslide occurred in Wuxia, one of the famous Three Gorges of the Yangtze River^3^, there is a landslide of anti-inclined slope interbedded with soft and hard rock strata in Fushun west open-pit mine^4^.

Scholars have done a lot of useful research work on the catastrophic instability of soft–hard-interbedded anti-inclined rock slope. The nonlinear finite element method of two dimensions was used to analysis a highway slope in southwest area of China, owing to the discrepant weathering and the lower soft rock mass has some notch, so the collapse and the cracks was been seen in the upper hard rock mass^5^. Gongjiafang slope #2 is a anti-inclined slope consisted of thick/thin and soft/hard rocks layers at Wuxia Gorge section in the Three Gorges Reservoir area. The rock structures of this anti-inclined slope were investigated and its deformation process and failure mechanism influenced by rising water level of the reservoir were analyzed^6^. The deep and steep river are the key reasons to induct bending deformation onto the anti-inclined slope in the Wuxia Gorge area.

Based on the centrifugal model test and discrete element numerical simulation, the toppling process of soft–hard-interbedded toppling slopes and the failure mechanism under external disturbance were analyzed, the toppling process can be divided into three stages: initial deformation, steady deformation, and unstable failure^7^. According to centrifuge model test, the differences of toppling deformation and failure modes between the anti-inclined soft–hard-interbedded rock slopes and the anti-inclined layered rock slopes are studied, the existence of the soft rock also has influence on the ultimate bearing capacity and the toppling deformation degree of the slope, varying with the layer thickness ratio of the soft rock to the hard rock^8^. Three examples of rock slopes interbedded by hard and soft layers in the southwestern calcareous area of China were presented^9^. The influence of the anchorage angle and finding the most suitable anchoring method for a soft–hard-interbedded toppling deformed rock mass were studied, an anchorage angle of 45° is optimal^10^. The evolution process, deformation law, and failure mechanism of the Muzhailing Tunnel excavated in soft–hard-interbedded rock mass were studied^11^. The bending and toppling evaluation process, failure mechanisms of the soft and hard rock slopes, in the southwest stope of Taiping Mining, Inner Mongolia, were studied and summarized^12^.

There are many research achievements on stability analysis of slopes. A jointly distributed random variables method was used for reliability assessment of pseudo-static stability of rock slopes, and the conclusion is that the friction angle of sliding surface is the most effective parameter in rock slope stability with plane sliding^13^. The sequential compounding method was used for system reliability analysis of rock wedge stability considering correlated failure modes^14^, and the 3-D system probability of failure surface was presented and the probabilistic model was developed to evaluate the rock slope probability of failure^15^. A simplified semi-distinct element algorithm for discontinuous rock slopes under toppling instability assessment based on block theory was presented, using the modified second-order reliability with first-order efficiency for the reliability method^16^. Combined with a specific case, a fuzzy expert decision‑making system for rock slope block‑toppling modeling and assessment^17^, the Shear Strength Reduction technique was used for numerical analysis and stability assessment of complex secondary toppling failures^18^. The analytical solutions that incorporates the kinematic mechanisms of the jointed rock slope under the influence of groundwater and stabilizing the lowermost block subjected to slide head toppling were derived based on the limit equilibrium^19^. On the basis of the physical model testing, an equation to calculate the stability coefficient under flexural toppling was established with the limit equilibrium method and cantilever beam theory, and the most dangerous potential sliding surface is identified^20^.

A three‑dimensional discontinuous deformation analysis method was conducted to study the toppling failure mechanisms of hard rock slopes by introducing the global contact theory^21^. Distinct element method and finite element method were numerical simulation of slide-toe-toppling failure which is a kind of secondary toppling failures^22^. The numerical method discontinuous deformation analysis was used to study the supporting mechanism against flexural toppling failure, the lack of the support from slope toe is the crucial factor inducing the flexural toppling failure^23^. The centrifuge model was used to elucidate the failure mechanism of block-flexure toppling failure^24^. toppling-slumping failure can occur due to the shear failure of the weak horizontal soil/rock layer or as a result of differential settlement of the vertical rock columns within the continuous horizontal soil/rock layer or as a combination of the two mechanisms^25^. In western China, the steep-dip anaclinal metamorphic soft or soft–hard-interbedded strata with near parallel strikes in the river channel, V-shaped deeply incised river channels, and convex slopes are favorable conditions for the formation of deep-seated toppling deformations^26^.

At present, the main research methods on the slopes with interbeddings of soft and hard rocks include physical experiment method and numerical simulation method. According to the experimental phenomena and results, the formation of this kind of landslide is qualitatively described. The existing research results fail to reveal the internal mechanical mechanism of the instability and failure for the anti-inclined slope with interbeddings of soft and hard rocks, the transmission mechanical mechanism of slope rock stratum interaction force has not be established, the theoretical mechanical method of stability analysis for the soft–hard-interbedded anti-inclined rock slope needs to be proposed urgently.

In this study, the geomechanical model of the anti-inclined slope with interbeddings of soft and hard rocks is established. Then, based on the limit equilibrium theory, the transmission mechanism of interaction force between rock layers is analyzed. Thirdly, the theoretical method for calculating the overall stability of soft–hard-interbedded anti-inclined rock slope is proposed. Finally, the experimental research results of other scholars are compared with the theoretical computational results proposed in this study. Compared with the physical test method and numerical method involved in the existing research results, the theoretical computational method proposed in this study is simpler and more convenient.

## Geological–mechanical model

**Figure 1:**
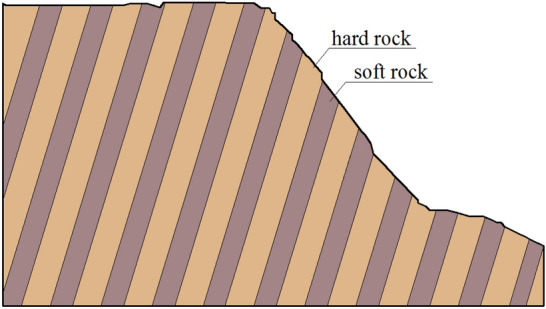
Schematic diagram for initial state of soft–hard-interbedded anti-inclined rock slope.

**Figure 2:**
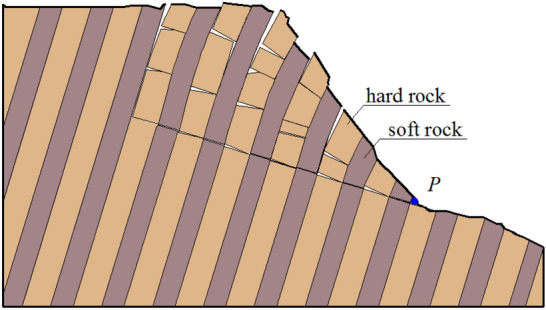
Schematic diagram for deformation and failure state of soft–hard-interbedded anti-inclined rock slope.

According to the analysis of a large number of engineering cases, it can be concluded that the thickness of hard rock stratum is close to that of soft rock stratum, and the thickness of hard rock stratum is slightly greater than that of soft rock stratum, in the anti-inclined slope with interbeddings of soft and hard rock strata. If the thickness of soft rock stratum is much smaller than that of hard rock stratum, it is called "soft interlayer in rock mass", which is significantly different from "soft–hard-interbedded rock mass". In addition, the thickness of hard rock stratum and soft rock stratum should not be too small, generally speaking, 3–8 groups of soft and hard alternating rock strata form a typical soft–hard-interbedded anti-inclined rock slope. If the thickness of hard rock stratum and soft rock stratum are both very small, there are a lot of alternating soft and hard rock strata, forming a thin anti-inclined slope. When the thickness of hard rock stratum and soft rock stratum in the anti-inclined slope is very large, the number of soft and hard alternating rock strata are very small, because the hard rock stratum is thick, it is not easy to form toppling failure, generally, a rock cavity is formed in the soft rock stratum, resulting in collapse failure. Therefore, we can generalize the soft–hard-interbedded anti-inclined rock slope model. Figure 1 shows the initial state of slope, and Fig. 2 shows the deformation and failure states of slope. In this model, the soft rock stratum is not heavily weathered, So no rock cavities were formed. Moreover, because soft rock stratum can withstand more deformation, it will have fewer fractures than that of hard rock stratum.

**Figure 3:**
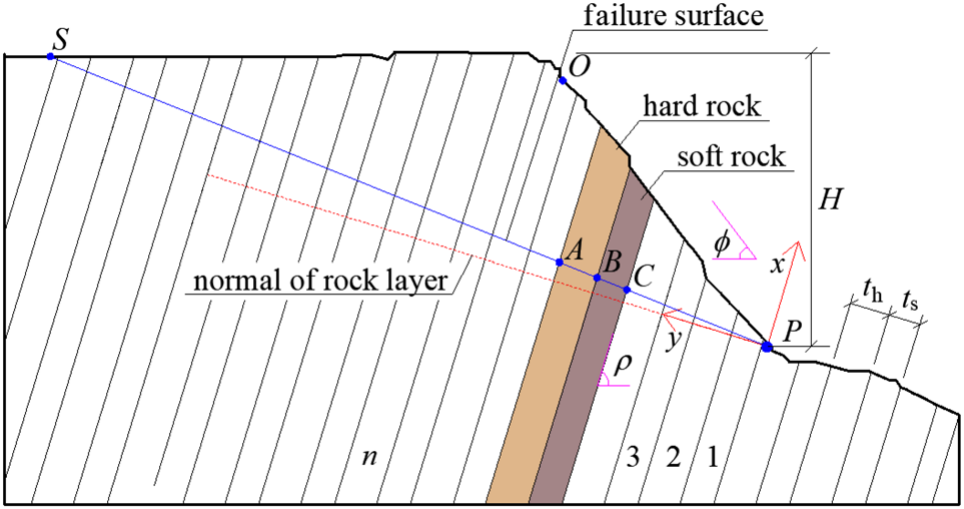
The analysis model of soft–hard-interbedded anti-inclined rock slope.

The analysis model of soft–hard-interbedded anti-inclined rock slope is shown in Fig. 3, the thickness of hard rock stratum is expressed as , the thickness of soft rock stratum is expressed as $t_{{\text{s}}}$, the dip angle of rock stratum is expressed as , the slope surface is approximately taken as a plane, the slope inclination is expressed as $\phi$, and the slope height is expressed as , the total vertical thickness of rock strata in part of slope body is expressed as $t_{z}$, the oblique length of surface on the slope is set to , $T = {{t_{{\text{z}}} } \mathord{\left/ {\vphantom {{t_{{\text{z}}} } {\sin (\pi - \rho - \phi )}}} \right. \kern-0pt} {\sin (\pi - \rho - \phi )}}$. It is assumed that each soft rock stratum is approximately equal in thickness, and each hard rock stratum is approximately equal in thickness. The rock stratum is numbered from bottom to top, with rock stratum 1 at the foot of the slope and rock stratum n at the top of the slope. The existing research work^27–31^ show that the failure surface of the overall anti inclined slope is planar or approximately planar in some areas of the rock strata, therefore, in the two-dimensional analysis, it can be assumed that the failure line of the slope is a straight line passing through the toe of the slope, the included angle between the straight line and the horizontal plane is , and the value range of $\omega^{j}$ is . The dip angle of failure surface for the slope is expressed as $\upsilon$, . For a specific slope, $\upsilon$ is a fixed value, by calculating multiple , the best $\upsilon$ is obtained.

**Figure 4:**
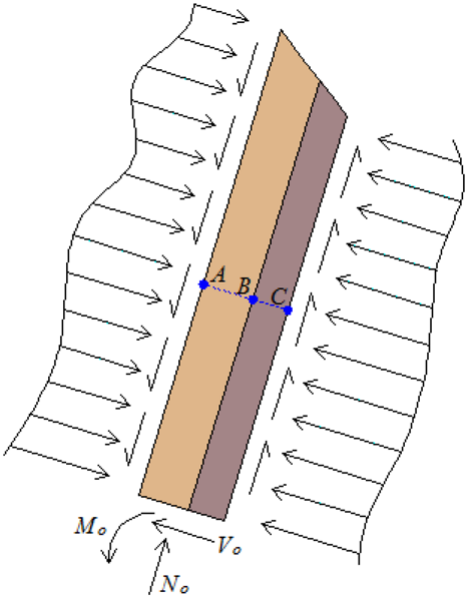
Stress state diagram of adjacent soft and hard rock stratum.

In Fig. 3, a rectangular coordinate system is established with the length direction of the rock stratum as the x-axis and the normal direction of the rock stratum as the y-axis. The failure cross section of each rock stratum is distributed near the y-axis. Take any adjacent soft and hard rock stratum in the slope as the analysis object, and its stress state is shown in Fig. 4.

According to the principle of limit equilibrium and the balance conditions of force and moment, the equilibrium equation of the possible toppling and sliding failure state of each rock stratum is established, and the interaction force between rock strata is calculated to determine the failure position of each rock stratum.

In order to simplify the calculation, the following assumptions are made:

1. The x-axis displacement of the neutral axis of the rock stratum is ignored, and the extrusion deformation in the y-axis direction is ignored;
2. It conforms to the assumption of plane section, that is, the plane perpendicular to the center line before deformation remains a plane after deformation (not necessarily perpendicular to the deflection curve);
3. The transverse shear deformation of rock stratum is not considered.

**Figure 5:**
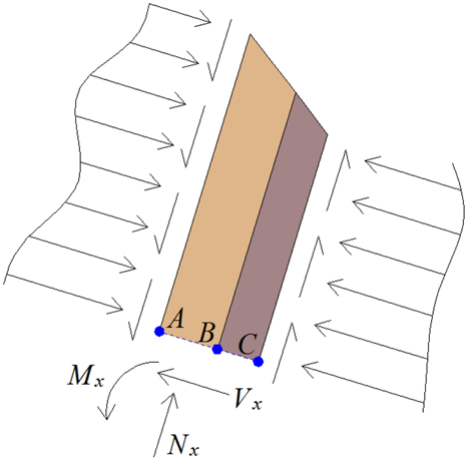
Stress state diagram of rock stratum unit above the failure surface.

The intersection of the failure line and the adjacent soft and hard rock strata in the slope is three points respectively: *A*, *B*, *C*. Further, the rock unit above the failure surface is taken as the research object, and its stress state is shown in Fig. 5. The upper surface of the hard rock stratum is subjected to the normal compressive stress and tangential friction of the overlying rock stratum. The lower surface of the soft rock stratum is subjected to supporting force and friction force from the underlying rock stratum. Shear force, bending moment and axial pressure are formed at the failure section $ABC$ of soft and hard rock stratum.

In order to analyze the stability of soft–hard-interbedded anti-inclined rock slope, it is also necessary to obtain relevant geometric parameters, physical and mechanical parameters. The gravity of soft rock stratum is expressed as $\gamma_{{\text{s}}}$. The tensile strength, internal friction angle and cohesion on the cross section of soft rock stratum are set as $\sigma_{{\text{s}}}$, $\varphi_{{\text{s}}}$ and $c_{{\text{s}}}$, respectively. The gravity of hard rock stratum is expressed as $\gamma_{{\text{h}}}$. The tensile strength, internal friction angle and cohesion on the cross section of hard rock stratum are set as $\sigma_{{\text{h}}}$, $\varphi_{{\text{h}}}$ and $c_{{\text{h}}}$, respectively. The cohesion and internal friction angle between soft rock stratum and hard rock stratum are expressed as $c_{{{\text{hs}}}}$ and $\varphi_{{{\text{hs}}}}$, respectively.

For a certain slope, the vertical line $PS$ of the rock stratum can be made through point $P$ at the slope toe. Point $S$ is another intersection for the normal of the rock stratum and the slope surface. The length of any rock stratum $i$ above the $PS$ line is expressed as $L_{i}$. The overall penetrating failure line $PS^{\prime}$ of instability in the slope rock strata is also a straight line passing through point $P$ at the slope toe. The dip angle of line $PS^{\prime}$ is expressed as $\omega^{j}$, and $\omega^{j} \in [90^\circ - \rho ,\phi ]$. Through the geometric relationship, the length $l_{i}^{j}$ of each rock stratum above the failure section can be obtained, and the length of the thickness centerline of each rock stratum is taken as the standard for length calculation. The vertical distance between the center line in the thickness of rock stratum $i$ and point $P$ is recorded as $t_{zi}$, so the gravity $W_{i}^{j}$ of each rock stratum above the failure section can be calculated.

1 $$l_{i}^{j} = t_{zi} \left[ {\tan \left( {\phi + \rho - 0.5\pi } \right) - \tan \left( {\omega^{j} + \rho - 0.5\pi } \right)} \right]$$

2 $$W_{i}^{j} = \gamma_{{\text{s}}} l_{i}^{j} t_{{\text{s}}} \quad {\text{or}}\quad W_{i}^{j} = \gamma_{{\text{h}}} l_{i}^{j} t_{{\text{h}}}$$

## Stress analysis of rock stratum

In this kind of slope, due to the gradual formation of the inclined slope, some areas of the rock stratum are in the free state, resulting in the unbalanced stress state of the rock stratum in the slope. The essence of the failure for the anti-inclined rock stratum is the bending and shear deformation failure of the rock stratum under the action of the overlying load and its own gravity. Therefore, the overlying load and its own gravity are the main factors leading to the failure of the rock stratum, belonging to the active force. The shear resistance, bending resistance of the rock stratum itself and the supporting force of the lower rock stratum belong to passive resistance. Therefore, when analyzing the stress of the rock stratum, it should be analyzed from top to bottom.

Beyond a certain range away from the top of the slope, there is a certain rock stratum *n* + 1 in the back direction of the slope, which is in the state of stress balance. Because the shear and flexural strength of this rock stratum can resist the shear stress and flexural tensile stress caused by eccentric gravity, it is in a stable state, its force on the underlying rock stratum (i.e. right rock stratum *n*) is zero. The rock stratum *n* on the right side has experienced weathering, earthquake, etc., formed toppling failure or shear failure under its own gravity, and transmitted its own gravity to rock stratum *n*-1, so that the loads between rock strata are transmitted to each other until the slope toe. When the transmission force is positive, it is proved that the slope has been damaged. In practical engineering, this situation is only applicable to the inverse analysis of the damaged slope.

When analyzing the stability of the anti-inclined rock slope which has not been formed overall failure, it is mainly to compare and analyze the transmission of force between rock strata in the rock slope and the effective bending and shear resistance of the rock strata, so as to determine the possibility of the overall failure for the anti-inclined rock strata at the present stage or in the future.

### Determination of the first damaged rock stratum

The rock strata within a certain range behind the slope may form an overall failure surface with the rock strata of the slope. In Fig. 3, the intersection of the rock stratum normal (y-axis) and the rock stratum surface behind the slope is point $S$. Therefore, for the anti-inclined rock stratum within the $OS$ range, from far to near, the stability of each rock stratum under the action of gravity needs to be analyzed, and its force on the underlying rock stratum should be calculated.

**Figure 6:**
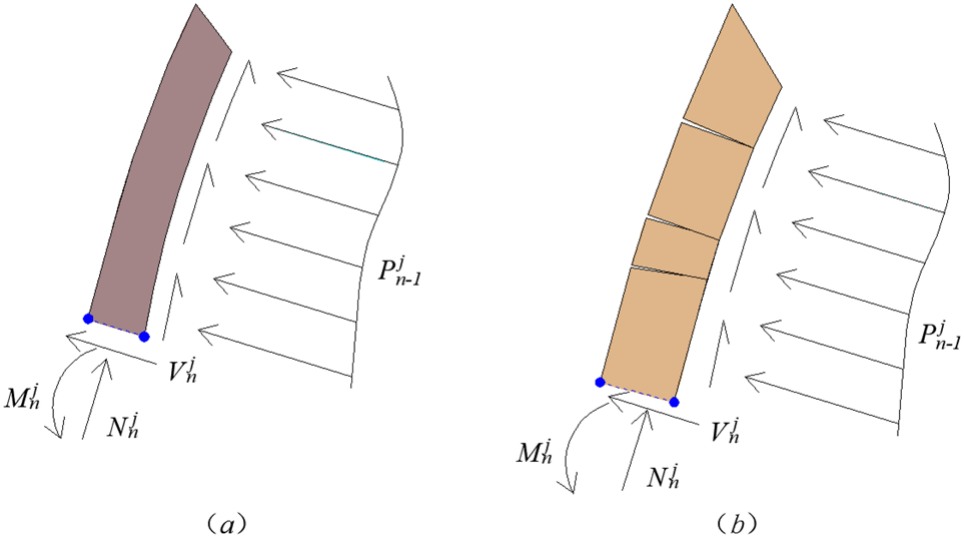
The stress analysis diagram for the first damaged rock stratum.

Figure 6 is the stress analysis diagram for the first damaged rock stratum. Figure 6a shows that the soft rock stratum forms shear failure under the action of gravity, and acts on the underlying rock stratum. Figure 6b shows the bending and toppling failure of hard rock stratum under the action of gravity, and acts on the underlying rock stratum.

When it is known that there is a potential failure section in the whole slope, if the rock stratum $n$ behind the slope is determined, it means that the uppermost and leftmost failure rock stratum of the overall failure body for the slope are determined. The force of the rock stratum $n$ on the underlying rock stratum $n - 1$ is set as $P_{n - 1}^{j}$, and the supporting force of the rock stratum $n - 1$ on the rock stratum $n$ is also set as $P_{n - 1}^{j}$. $\eta_{i}$ represents the coefficient of force for the normal thrust transmitted from rock stratum $i + 1$ to rock stratum $i$. When rock stratum $i + 1$ is a soft rock stratum, its own shaping deformation is large. After forming shear failure along the failure section, the length $l_{i + 1}^{j}$ of rock stratum above the failure section, slides to the underlying rock stratum $i$ , and the point of resultant force is near the middle point of the length $l_{i + 1}^{j}$, so $\eta_{i} { = }0.5$. When rock stratum $i + 1$ is a hard rock stratum, its own shaping deformation is small. After toppling failure is formed along the failure section, the top of the length $l_{i + 1}^{j}$ above the failure section, acts on the underlying rock stratum $i$, and the point of resultant force is near the other end of the length $l_{i + 1}^{j}$ above the failure section, so $\eta_{i} { = }1.0$. Of course, soft rock stratum can also form toppling failure, and hard rock stratum can also form sliding failure, it's not absolute.

1. When the first damaged rock stratum $n$ behind the slope may be soft rock stratum (as shown in Fig. 6a).
  1. It is assumed that the rock stratum $n$ will slide when it is damaged, its thrust action transmitted to the next rock stratum is $\left( {P_{n - 1}^{j} } \right)^{\prime }$, $\eta^{\prime}_{{n{ - }1}} = 0.5$,
    3 $$\left( {P_{n - 1}^{j} } \right)^{\prime } = \frac{{\left( {\gamma_{{\text{s}}} l_{n}^{j} t_{{\text{s}}} \cos \rho } \right) - c_{{\text{s}}} t_{{\text{s}}} - \left( {\gamma_{{\text{s}}} l_{n}^{j} t_{{\text{s}}} {\text{sin}}\rho } \right)\tan \varphi_{{\text{s}}} + \left( {c_{{{\text{hs}}}} l_{n}^{j} } \right)\tan \varphi_{{\text{s}}} }}{{1 - \tan \varphi_{{{\text{hs}}}} \cdot \tan \varphi_{{\text{s}}} }}$$
    At the same time, the safety factor of sliding failure for rock stratum $n$ is defined as
    4 $$\left( {f_{{{\text{s}}n}}^{j} } \right)_{s} = \frac{{c_{{\text{s}}} t_{{\text{s}}} + \left( {\gamma_{{\text{s}}} t_{{\text{s}}} l_{n}^{j} {\text{sin}}\rho } \right)\tan \varphi_{{\text{s}}} }}{{\left( {\gamma_{{\text{s}}} l_{n}^{j} t_{{\text{s}}} \cos \rho } \right) + \left( {c_{{{\text{hs}}}} l_{n}^{j} } \right)\tan \varphi_{{\text{s}}} }}$$
  2. It is assumed that the rock stratum $n$ will topple and fall when it is damaged, its thrust action transmitted to the next rock stratum is $\left( {P_{n - 1}^{j} } \right)^{\prime \prime }$, $\eta^{\prime\prime}_{{n{ - }1}} = 1.0$,
    5 $$\left( {P_{n - 1}^{j} } \right)^{\prime \prime } = \frac{{0.5\gamma_{{\text{s}}} \left( {l_{n}^{j} } \right)^{2} t_{{\text{s}}} \cos \rho - 0.5\gamma_{{\text{s}}} t_{{\text{s}}}^{2} l_{n}^{j} {\text{sin}}\rho - 0.5\sigma_{{\text{s}}} t_{{\text{s}}}^{{2}} k_{{\text{s}}} }}{{\eta_{n - 1} l_{n}^{j} }}$$
    At the same time, the safety factor of toppling failure for rock stratum $n$ is defined as
    6 $$\left( {f_{{{\text{s}}n}}^{j} } \right)_{t} = \frac{{0.5\gamma_{{\text{s}}} t_{{\text{s}}}^{{2}} l_{n}^{j} {\text{sin}}\rho + 0.5\sigma_{{\text{s}}} t_{{\text{s}}}^{{2}} k_{{\text{s}}} }}{{0.5\gamma_{{\text{s}}} \left( {l_{n}^{j} } \right)^{2} t_{{\text{s}}} \cos \rho }}$$
  3. If $\left( {P_{n - 1}^{j} } \right)^{\prime } > 0$, and $\left( {P_{n - 1}^{j} } \right)^{\prime \prime } > 0$, the sizes of $\left( {f_{{{\text{s}}n}}^{j} } \right)_{s}$ and $\left( {f_{{{\text{s}}n}}^{j} } \right)_{t}$ need to be compared.
    ① When $$\left( {f_{{{\text{s}}n}}^{j} } \right)_{s} < \left( {f_{{{\text{s}}n}}^{j} } \right)_{t}$$, then $$P_{n - 1}^{j} = \left( {P_{n - 1}^{j} } \right)^{\prime }$$, $$f_{{{\text{s}}n}}^{j} = \left( {f_{{{\text{s}}n}}^{j} } \right)_{s}$$, $$\eta_{{n{ - }1}} = \eta^{\prime}_{{n{ - }1}} = 0.5$$.
    ② When $$\left( {f_{{{\text{s}}n}}^{j} } \right)_{s} > \left( {f_{{{\text{s}}n}}^{j} } \right)_{t}$$, then $$P_{n - 1}^{j} = \left( {P_{n - 1}^{j} } \right)^{\prime \prime }$$, $$f_{{{\text{s}}n}}^{j} = \left( {f_{{{\text{s}}n}}^{j} } \right)_{t}$$, $$\eta_{{n{ - }1}} = \eta^{\prime\prime}_{{n{ - }1}} = 1$$.
  4. If $\left( {P_{n - 1}^{j} } \right)^{\prime } > 0$, and $\left( {P_{n - 1}^{j} } \right)^{\prime \prime } < 0$, then $P_{n - 1}^{j} = \left( {P_{n - 1}^{j} } \right)^{\prime }$, $f_{{{\text{s}}n}}^{j} = \left( {f_{{{\text{s}}n}}^{j} } \right)_{s}$, $\eta_{{n{ - }1}} = \eta^{\prime}_{{n{ - }1}} = 0.5$.
  5. If $\left( {P_{n - 1}^{j} } \right)^{\prime } < 0$, and $\left( {P_{n - 1}^{j} } \right)^{\prime \prime } > 0$, then $P_{n - 1}^{j} = \left( {P_{n - 1}^{j} } \right)^{\prime \prime }$, $f_{{{\text{s}}n}}^{j} = \left( {f_{{{\text{s}}n}}^{j} } \right)_{t}$, $\eta_{{n{ - }1}} = \eta^{\prime\prime}_{{n{ - }1}} = 1$.
  6. If $\left( {P_{n - 1}^{j} } \right)^{\prime } < 0$, and $\left( {P_{n - 1}^{j} } \right)^{\prime \prime } < 0$, then $P_{n - 1}^{j} = 0$, $f_{{{\text{s}}n}}^{j} = \min \left( {\left( {f_{{{\text{s}}n}}^{j} } \right)_{s} ,\left( {f_{{{\text{s}}n}}^{j} } \right)_{t} } \right)$, $\eta_{{n{ - }1}} = 0$.
2. When the first damaged rock stratum $n$ behind the slope may be hard rock stratum (as shown in Fig. 6b).
  1. It is assumed that the rock stratum $n$ will slide when it is damaged, its thrust action transmitted to the next rock stratum is $\left( {P_{n - 1}^{j} } \right)^{\prime \prime \prime }$, $\eta^{\prime \prime\prime}_{{n{ - }1}} = 0.5$,
    7 $$\left( {P_{n - 1}^{j} } \right)^{\prime \prime \prime } = \frac{{\left( {\gamma_{{\text{h}}} l_{n}^{j} t_{{\text{h}}} \cos \rho } \right) - c_{{\text{h}}} t_{{\text{h}}} - \left( {\gamma_{{\text{h}}} l_{n}^{j} t_{{\text{h}}} {\text{sin}}\rho } \right)\tan \varphi_{{\text{h}}} + \left( {c_{{{\text{hs}}}} l_{n}^{j} } \right)\tan \varphi_{{\text{h}}} }}{{1 - \tan \varphi_{{{\text{hs}}}} \cdot \tan \varphi_{{\text{h}}} }}$$
    At the same time, the safety factor of sliding failure for rock stratum $n$ is defined as
    8 $$\left( {f_{{{\text{hn}}}}^{j} } \right)_{s} = \frac{{c_{{\text{h}}} t_{{\text{h}}} + \left( {\gamma_{{\text{h}}} t_{{\text{h}}} l_{n}^{j} {\text{sin}}\rho } \right)\tan \varphi_{{\text{h}}} }}{{\left( {\gamma_{{\text{h}}} l_{n}^{j} t_{{\text{h}}} \cos \rho } \right) + \left( {c_{{{\text{hs}}}} l_{n}^{j} } \right)\tan \varphi_{{\text{h}}} }}$$
  2. It is assumed that the rock stratum $n$ will topple and fall when it is damaged, its thrust action transmitted to the next rock stratum is $\left( {P_{n - 1}^{j} } \right)^{\prime \prime \prime \prime }$, $\eta^{\prime\prime \prime\prime}_{{n{ - }1}} = 1.0$,
    9 $$\left( {P_{n - 1}^{j} } \right)^{\prime \prime \prime \prime } = \frac{{0.5\gamma_{{\text{h}}} \left( {l_{n}^{j} } \right)^{2} t_{{\text{h}}} \cos \rho - 0.5\gamma_{{\text{h}}} t_{{\text{h}}}^{2} l_{n}^{j} {\text{sin}}\rho - 0.5\sigma_{{\text{h}}} t_{{\text{h}}}^{{2}} k_{{\text{h}}} }}{{\eta_{n - 1} l_{n}^{j} }}$$
    At the same time, the safety factor of toppling failure for rock stratum $n$ is defined as
    10 $$\left( {f_{{{\text{h}}n}}^{j} } \right)_{t} = \frac{{0.5\gamma_{{\text{h}}} t_{{\text{h}}}^{{2}} l_{n}^{j} {\text{sin}}\rho + 0.5\sigma_{{\text{h}}} t_{{\text{h}}}^{{2}} k_{{\text{h}}} }}{{0.5\gamma_{{\text{h}}} \left( {l_{n}^{j} } \right)^{2} t_{{\text{h}}} \cos \rho }}$$
  3. If $\left( {P_{n - 1}^{j} } \right)^{\prime \prime \prime } > 0$, and $\left( {P_{n - 1}^{j} } \right)^{\prime \prime \prime \prime } > 0$, the sizes of $\left( {f_{{{\text{h}}n}}^{j} } \right)_{s}$ and $\left( {f_{{{\text{h}}n}}^{j} } \right)_{t}$ need to be compared.
    ① When $$\left( {f_{{{\text{h}}n}}^{j} } \right)_{s} < \left( {f_{{{\text{h}}n}}^{j} } \right)_{t}$$, then $$P_{n - 1}^{j} = \left( {P_{n - 1}^{j} } \right)^{\prime \prime \prime }$$, $$f_{{{\text{h}}n}}^{j} = \left( {f_{{{\text{h}}n}}^{j} } \right)_{s}$$, $$\eta_{{n{ - }1}} = \eta^{\prime \prime\prime}_{{n{ - }1}} = 0.5$$.
    ② When $$\left( {f_{{{\text{h}}n}}^{j} } \right)_{s} > \left( {f_{{{\text{h}}n}}^{j} } \right)_{t}$$, then $$P_{n - 1}^{j} = \left( {P_{n - 1}^{j} } \right)^{\prime \prime \prime \prime }$$, $$f_{{{\text{h}}n}}^{j} = \left( {f_{{{\text{h}}n}}^{j} } \right)_{t}$$, $$\eta_{{n{ - }1}} = \eta^{\prime\prime \prime\prime}_{{n{ - }1}} = 1$$.
  4. If $\left( {P_{n - 1}^{j} } \right)^{\prime \prime \prime } > 0$, and $\left( {P_{n - 1}^{j} } \right)^{\prime \prime \prime \prime } < 0$, then $P_{n - 1}^{j} = \left( {P_{n - 1}^{j} } \right)^{\prime \prime \prime }$, $f_{{{\text{h}}n}}^{j} = \left( {f_{{{\text{h}}n}}^{j} } \right)_{s}$, $\eta_{{n{ - }1}} = \eta^{\prime \prime\prime}_{{n{ - }1}} = 0.5$.
  5. If $\left( {P_{n - 1}^{j} } \right)^{\prime \prime \prime } < 0$, and $\left( {P_{n - 1}^{j} } \right)^{\prime \prime \prime \prime } > 0$, then $P_{n - 1}^{j} = \left( {P_{n - 1}^{j} } \right)^{\prime \prime \prime \prime }$, $f_{{{\text{h}}n}}^{j} = \left( {f_{{{\text{h}}n}}^{j} } \right)_{t}$, $\eta_{{n{ - }1}} = \eta^{\prime\prime \prime\prime}_{{n{ - }1}} = 1$.
  6. If $\left( {P_{n - 1}^{j} } \right)^{\prime \prime \prime } < 0$, and $\left( {P_{n - 1}^{j} } \right)^{\prime \prime \prime \prime } < 0$, then $P_{n - 1}^{j} = 0$, $f_{{{\text{h}}n}}^{j} = \min \left( {\left( {f_{{{\text{h}}n}}^{j} } \right)_{s} ,\left( {f_{{{\text{h}}n}}^{j} } \right)_{t} } \right)$, $\eta_{{n{ - }1}} = 0$.

### Stress analysis of arbitrary rock stratum in the middle of anti-inclined slope

**Figure 7:**
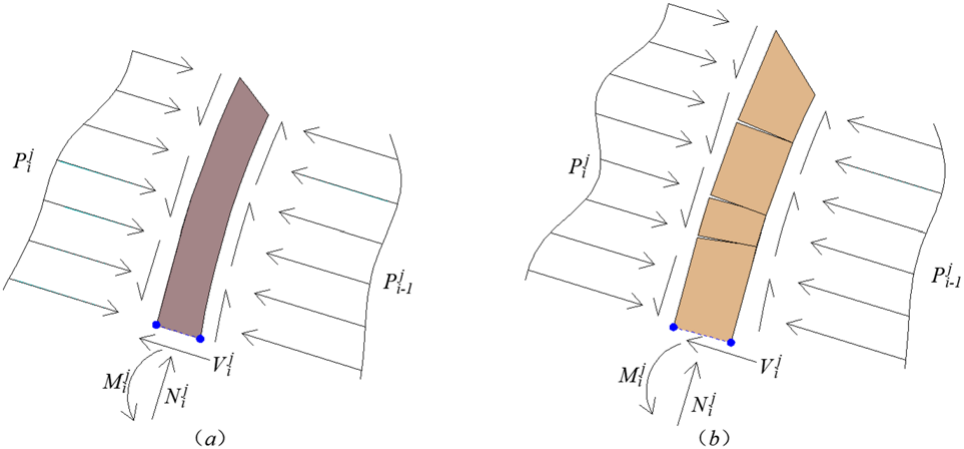
The stress analysis diagram of arbitrary stratum $i$ in the middle part of anti-inclined slope.

After the top damaged rock stratum is determined, the damage of each rock stratum in the middle of the anti-inclined rock slope needs to be determined in turn. Figure 7 is the corresponding stress analysis diagram. In the middle part of the anti-inclined rock slope, the interaction force between any rock stratum $i$ and the underlying rock stratum $i - 1$ is $P_{i - 1}^{j}$.

1. When the arbitrary stratum $i$, in the middle part of the anti-inclined rock slope, may be soft rock stratum (as shown in Fig. 7a).
  1. It is assumed that the rock stratum $i$ will slide when it is damaged, its thrust action transmitted to the next rock stratum is $\left( {P_{i - 1}^{j} } \right)^{\prime }$, $\eta^{\prime}_{{i{ - }1}} = 0.5$,
    11 $$\left( {P_{i - 1}^{j} } \right)^{\prime } = \frac{{P_{i}^{j} + \gamma_{{\text{s}}} l_{i}^{j} t_{{\text{s}}} \cos \rho - c_{{\text{s}}} t_{{\text{s}}} - \left( {P_{i}^{j} \tan \varphi_{{{\text{hs}}}} + \gamma_{{\text{s}}} t_{{\text{s}}} l_{i}^{j} {\text{sin}}\rho } \right)\tan \varphi_{{\text{s}}} }}{{1 - \tan \varphi_{{{\text{hs}}}} \cdot \tan \varphi_{{\text{s}}} }}$$
    At the same time, the safety factor of sliding failure for rock stratum $i$ is defined as
    12 $$\left( {f_{{{\text{s}}i}}^{j} } \right)_{s} = \frac{{c_{{\text{s}}} t_{{\text{s}}} + \left( {P_{i}^{j} \tan \varphi_{{{\text{hs}}}} + \gamma_{{\text{s}}} t_{{\text{s}}} l_{i}^{j} {\text{sin}}\rho } \right)\tan \varphi_{{\text{s}}} }}{{P_{i}^{j} + \gamma_{{\text{s}}} l_{i}^{j} t_{{\text{s}}} \cos \rho }}$$
  2. It is assumed that the rock stratum $i$ will topple and fall when it is damaged, its thrust action transmitted to the next rock stratum is $\left( {P_{i - 1}^{j} } \right)^{\prime \prime }$, $\eta^{\prime\prime}_{{i{ - }1}} = 1.0$,
    13 $$\left( {P_{i - 1}^{j} } \right)^{\prime \prime } = \frac{{\eta_{i} P_{i}^{j} l_{i}^{j} + 0.5\gamma_{{\text{s}}} \left( {l_{i}^{j} } \right)^{2} t_{{\text{s}}} \cos \rho - 0.5\gamma_{{\text{s}}} t_{{\text{s}}}^{2} l_{i}^{j} {\text{sin}}\rho - P_{i}^{j} t_{{\text{s}}} \tan \varphi_{{{\text{hs}}}} - 0.5\sigma_{{\text{s}}} t_{{\text{s}}}^{{2}} k_{{\text{s}}} - c_{{{\text{hs}}}} l_{i}^{j} t_{{\text{s}}} }}{{\eta_{i - 1} l_{i}^{j} }}$$
    At the same time, the safety factor of toppling failure for rock stratum $i$ is defined as
    14 $$\left( {f_{{{\text{s}}i}}^{j} } \right)_{t} = \frac{{0.5\gamma_{{\text{s}}} t_{{\text{s}}}^{{2}} l_{i}^{j} {\text{sin}}\rho + 0.5\sigma_{{\text{s}}} t_{{\text{s}}}^{{2}} k_{{\text{s}}} + P_{i}^{j} t_{{\text{s}}} \tan \varphi_{{{\text{hs}}}} + c_{{{\text{hs}}}} l_{i}^{j} t_{{\text{s}}} }}{{\eta_{i} P_{i}^{j} l_{i}^{j} + 0.5\gamma_{{\text{s}}} \left( {l_{i}^{j} } \right)^{2} t_{{\text{s}}} \cos \rho }}$$
  3. If $\left( {P_{i - 1}^{j} } \right)^{\prime } > 0$, and $\left( {P_{i - 1}^{j} } \right)^{\prime \prime } > 0$, the sizes of $\left( {f_{{{\text{s}}i}}^{j} } \right)_{s}$ and $\left( {f_{{{\text{s}}i}}^{j} } \right)_{t}$ need to be compared.
    ① When $$\left( {f_{{{\text{s}}i}}^{j} } \right)_{s} < \left( {f_{{{\text{s}}i}}^{j} } \right)_{t}$$, then $$P_{i - 1}^{j} = \left( {P_{i - 1}^{j} } \right)^{\prime }$$, $$f_{{{\text{s}}i}}^{j} = \left( {f_{{{\text{s}}i}}^{j} } \right)_{s}$$, $$\eta_{{i{ - }1}} = \eta^{\prime}_{{i{ - }1}} = 0.5$$.
    ② When $$\left( {f_{{{\text{s}}i}}^{j} } \right)_{s} > \left( {f_{{{\text{s}}i}}^{j} } \right)_{t}$$, then $$P_{i - 1}^{j} = \left( {P_{i - 1}^{j} } \right)^{\prime \prime }$$, $$f_{{{\text{s}}i}}^{j} = \left( {f_{{{\text{s}}i}}^{j} } \right)_{t}$$, $$\eta_{{i{ - }1}} = \eta^{\prime\prime}_{{i{ - }1}} = 1.0$$.
  4. If $\left( {P_{i - 1}^{j} } \right)^{\prime } > 0$, and $\left( {P_{i - 1}^{j} } \right)^{\prime \prime } < 0$, then $P_{i - 1}^{j} = \left( {P_{i - 1}^{j} } \right)^{\prime }$, $f_{{{\text{s}}i}}^{j} = \left( {f_{{{\text{s}}i}}^{j} } \right)_{s}$, $\eta_{{i{ - }1}} = \eta^{\prime}_{{i{ - }1}} = 0.5$.
  5. If $\left( {P_{i - 1}^{j} } \right)^{\prime } < 0$, and $\left( {P_{i - 1}^{j} } \right)^{\prime \prime } > 0$, then $P_{i - 1}^{j} = \left( {P_{i - 1}^{j} } \right)^{\prime \prime }$, $f_{{{\text{s}}i}}^{j} = \left( {f_{{{\text{s}}i}}^{j} } \right)_{t}$, $\eta_{{i{ - }1}} = \eta^{\prime\prime}_{{i{ - }1}} = 1.0$.
  6. If $\left( {P_{i - 1}^{j} } \right)^{\prime } < 0$, and $\left( {P_{i - 1}^{j} } \right)^{\prime \prime } < 0$, then $P_{i - 1}^{j} = 0$, $f_{{{\text{s}}i}}^{j} = \min \left( {\left( {f_{{{\text{s}}i}}^{j} } \right)_{s} ,\left( {f_{{{\text{s}}i}}^{j} } \right)_{t} } \right)$, $\eta_{{i{ - }1}} = 0$.

(2) When the arbitrary stratum $i$, in the middle part of the anti-inclined rock slope, may be hard rock stratum (as shown in Fig. 7b).
  1. It is assumed that the rock stratum $i$ will slide when it is damaged, its thrust action transmitted to the next rock stratum is $\left( {P_{i - 1}^{j} } \right)^{\prime \prime \prime }$, $\eta^{\prime \prime\prime}_{{i{ - }1}} = 0.5$,
    15 $$\left( {P_{i - 1}^{j} } \right)^{\prime \prime \prime } = \frac{{P_{i}^{j} + \gamma_{{\text{h}}} l_{i}^{j} t_{{\text{h}}} \cos \rho - c_{{\text{h}}} t_{{\text{h}}} - \left( {P_{i}^{j} \tan \varphi_{{{\text{hs}}}} + \gamma_{{\text{h}}} t_{{\text{h}}} l_{i}^{j} {\text{sin}}\rho } \right)\tan \varphi_{{\text{h}}} }}{{1 - \tan \varphi_{{{\text{hs}}}} \cdot \tan \varphi_{{\text{h}}} }}$$
    At the same time, the safety factor of sliding failure for rock stratum $i$ is defined as
    16 $$\left( {f_{{{\text{h}}i}}^{j} } \right)_{s} = \frac{{c_{{\text{h}}} t_{{\text{h}}} + \left( {P_{i}^{j} \tan \varphi_{{{\text{hs}}}} + \gamma_{{\text{h}}} t_{{\text{s}}} l_{i}^{j} {\text{sin}}\rho } \right)\tan \varphi_{{\text{h}}} }}{{P_{i}^{j} + \gamma_{{\text{h}}} l_{i}^{j} t_{{\text{h}}} \cos \rho }}$$
  2. It is assumed that the rock stratum $i$ will topple and fall when it is damaged, its thrust action transmitted to the next rock stratum is $\left( {P_{i - 1}^{j} } \right)^{\prime \prime \prime \prime }$, $\eta^{\prime\prime \prime\prime}_{{i{ - }1}} = 1.0$,
    17 $$\left( {P_{i - 1}^{j} } \right)^{\prime \prime \prime \prime } = \frac{{\eta_{i} P_{i}^{j} l_{i}^{j} + 0.5\gamma_{{\text{h}}} \left( {l_{i}^{j} } \right)^{2} t_{{\text{h}}} \cos \rho - 0.5\gamma_{{\text{h}}} t_{{\text{h}}}^{2} l_{i}^{j} {\text{sin}}\rho - P_{i}^{j} t_{{\text{h}}} \tan \varphi_{{{\text{hs}}}} - 0.5\sigma_{{\text{h}}} t_{{\text{h}}}^{{2}} k_{{\text{h}}} - c_{{{\text{hs}}}} l_{i}^{j} t_{{\text{h}}} }}{{\eta_{i - 1} l_{i}^{j} }}$$
    At the same time, the safety factor of toppling failure for rock stratum $i$ is defined as
    18 $$\left( {f_{{{\text{h}}i}}^{j} } \right)_{t} = \frac{{0.5\gamma_{{\text{h}}} t_{{\text{h}}}^{{2}} l_{i}^{j} {\text{sin}}\rho + 0.5\sigma_{{\text{h}}} t_{{\text{h}}}^{{2}} k_{{\text{h}}} + P_{i}^{j} t_{{\text{h}}} \tan \varphi_{{{\text{hs}}}} + c_{{{\text{hs}}}} l_{i}^{j} t_{{\text{h}}} }}{{\eta_{i} P_{i}^{j} l_{i}^{j} + 0.5\gamma_{{\text{h}}} \left( {l_{i}^{j} } \right)^{2} t_{{\text{h}}} \cos \rho }}$$
  3. If $\left( {P_{i - 1}^{j} } \right)^{\prime \prime \prime } > 0$, and $\left( {P_{i - 1}^{j} } \right)^{\prime \prime \prime \prime } > 0$, the sizes of $\left( {f_{{{\text{h}}i}}^{j} } \right)_{s}$ and $\left( {f_{{{\text{h}}i}}^{j} } \right)_{t}$ need to be compared.
    ① When $$\left( {f_{{{\text{h}}i}}^{j} } \right)_{s} < \left( {f_{{{\text{h}}i}}^{j} } \right)_{t}$$, then $$P_{i - 1}^{j} = \left( {P_{i - 1}^{j} } \right)^{\prime \prime \prime }$$, $$f_{{{\text{h}}i}}^{j} = \left( {f_{{{\text{h}}i}}^{j} } \right)_{s}$$, $$\eta_{{i{ - }1}} = \eta^{\prime \prime\prime}_{{i{ - }1}} = 0.5$$.
    ② When $$\left( {f_{{{\text{h}}i}}^{j} } \right)_{s} > \left( {f_{{{\text{h}}i}}^{j} } \right)_{t}$$, then $$P_{i - 1}^{j} = \left( {P_{i - 1}^{j} } \right)^{\prime \prime \prime \prime }$$, $$f_{{{\text{h}}i}}^{j} = \left( {f_{{{\text{h}}i}}^{j} } \right)_{t}$$, $$\eta_{{i{ - }1}} = \eta^{\prime\prime \prime\prime}_{{i{ - }1}} = 1.0$$.
  4. If $\left( {P_{i - 1}^{j} } \right)^{\prime \prime \prime } > 0$, and $\left( {P_{i - 1}^{j} } \right)^{\prime \prime \prime \prime } < 0$, then $P_{i - 1}^{j} = \left( {P_{i - 1}^{j} } \right)^{\prime \prime \prime }$, $f_{{{\text{h}}i}}^{j} = \left( {f_{{{\text{h}}i}}^{j} } \right)_{s}$, $\eta_{{i{ - }1}} = \eta^{\prime \prime\prime}_{{i{ - }1}} = 0.5$.
  5. If $\left( {P_{i - 1}^{j} } \right)^{\prime \prime \prime } < 0$, and $\left( {P_{i - 1}^{j} } \right)^{\prime \prime \prime \prime } > 0$, then $P_{i - 1}^{j} = \left( {P_{i - 1}^{j} } \right)^{\prime \prime \prime \prime }$, $f_{{{\text{h}}i}}^{j} = \left( {f_{{{\text{h}}i}}^{j} } \right)_{t}$, $\eta_{{i{ - }1}} = \eta^{\prime\prime \prime\prime}_{{i{ - }1}} = 1.0$.
  6. If $\left( {P_{i - 1}^{j} } \right)^{\prime \prime \prime } < 0$, and $\left( {P_{i - 1}^{j} } \right)^{\prime \prime \prime \prime } < 0$, then $P_{i - 1}^{j} = 0$, $f_{{{\text{h}}i}}^{j} = \min \left( {\left( {f_{{{\text{h}}i}}^{j} } \right)_{s} ,\left( {f_{{{\text{h}}i}}^{j} } \right)_{t} } \right)$, $\eta_{{i{ - }1}} = 0$.

### Stress analysis of last rock stratum at slope toe

**Figure 8:**
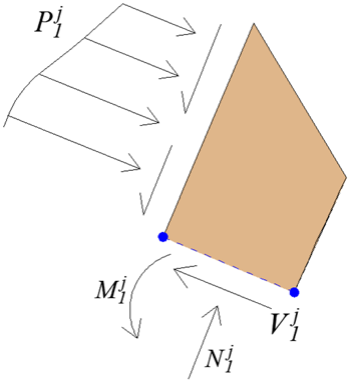
The stress analysis diagram of last rock stratum at slope toe.

The last rock stratum of rock at the slope toe should generally be hard rock, because soft rock is easy to form rock cavity due to excavation or weathering. Figure 8 is the stress analysis diagram of last rock stratum at slope toe. Under the load transmitted by the overlying rock strata, the last rock stratum may form toppling failure or shearing and sliding failure. Compared with the middle rock strata, the main difference is that the last rock stratum at the foot of the slope is no longer supported by the underlying rock stratum. The residual sliding thrust at the toe of the slope is $P_{0}^{j}$.

1. At the foot of the slope, it is assumed that the last rock stratum (stratum $1$) will slide when it is damaged, its residual sliding thrust is $\left( {P_{0}^{j} } \right)^{\prime }$,$\eta^{\prime}_{0} = 0.5$,
  19 $$\left( {P_{0}^{j} } \right)^{\prime } = P_{1}^{j} + \left( {\gamma_{{\text{h}}} l_{1}^{j} t_{{\text{h}}} \cos \rho } \right) - c_{{\text{h}}} t_{{\text{h}}} - \left( {\gamma_{{\text{h}}} l_{1}^{j} t_{{\text{h}}} \sin \rho } \right)\tan \varphi_{{\text{h}}}$$
  At the same time, the safety factor of sliding failure for rock stratum $1$ is defined as
  20 $$\left( {f_{{{\text{h}}1}}^{j} } \right)_{s} = \frac{{c_{{\text{h}}} t_{{\text{h}}} + \left( {\gamma_{{\text{h}}} l_{1}^{j} t_{{\text{h}}} \sin \rho } \right)\tan \varphi_{{\text{h}}} }}{{P_{1}^{j} + \left( {\gamma_{{\text{h}}} l_{1}^{j} t_{{\text{h}}} \cos \rho } \right)}}$$
2. At the foot of the slope, it is assumed that the last rock stratum (stratum $1$) will topple and fall when it is damaged, its residual sliding thrust is $\left( {P_{0}^{j} } \right)^{\prime \prime }$,$\eta^{\prime\prime}_{0} = 1.0$,
  21 $$\left( {P_{0}^{j} } \right)^{\prime \prime } = \frac{{\eta_{1} P_{1}^{j} l_{1}^{j} + 0.5\gamma_{{\text{h}}} \left( {l_{1}^{j} } \right)^{2} t_{{\text{h}}} \cos \rho - 0.5\gamma_{{\text{h}}} t_{{\text{h}}}^{2} l_{1}^{j} {\text{sin}}\rho - P_{1}^{j} t_{{\text{h}}} \tan \varphi_{{{\text{hs}}}} - 0.5\sigma_{{\text{h}}} t_{{\text{h}}}^{{2}} k_{{\text{h}}} - c_{{{\text{hs}}}} l_{1}^{j} t_{{\text{h}}} }}{{\eta_{0} l_{1}^{j} }}$$
  At the same time, the safety factor of toppling failure for rock stratum $1$ is defined as
  22 $$\left( {f_{{{\text{h}}1}}^{j} } \right)_{t} = \frac{{0.5\gamma_{{\text{h}}} t_{{\text{h}}}^{{2}} l_{1}^{j} {\text{sin}}\rho + 0.5\sigma_{{\text{h}}} t_{{\text{h}}}^{{2}} k_{{\text{h}}} + P_{1}^{j} t_{{\text{h}}} \tan \varphi_{{{\text{hs}}}} + c_{{{\text{hs}}}} l_{1}^{j} t_{{\text{h}}} }}{{\eta_{1} P_{1}^{j} l_{1}^{j} + 0.5\gamma_{{\text{h}}} \left( {l_{1}^{j} } \right)^{2} t_{{\text{h}}} \cos \rho }}$$
3. If $\left( {P_{0}^{j} } \right)^{\prime } > 0$, and $\left( {P_{0}^{j} } \right)^{\prime \prime } > 0$, the sizes of $\left( {f_{{{\text{h1}}}}^{j} } \right)_{s}$ and $\left( {f_{{{\text{h1}}}}^{j} } \right)_{t}$ need to be compared.
  1. When $$\left( {f_{{{\text{h1}}}}^{j} } \right)_{s} < \left( {f_{{{\text{h1}}}}^{j} } \right)_{t}$$, then $$P_{0}^{j} = \left( {P_{0}^{j} } \right)^{\prime }$$, $$f_{{{\text{h1}}}}^{j} = \left( {f_{{{\text{h1}}}}^{j} } \right)_{s}$$, $$\eta_{0} = \eta^{\prime}_{0} = 0.5$$.
  2. When $$\left( {f_{{{\text{h1}}}}^{j} } \right)_{s} > \left( {f_{{{\text{h1}}}}^{j} } \right)_{t}$$, then $$P_{0}^{j} = \left( {P_{0}^{j} } \right)^{\prime \prime }$$, $$f_{{{\text{h1}}}}^{j} = \left( {f_{{{\text{h1}}}}^{j} } \right)_{t}$$, $$\eta_{0} = \eta^{\prime\prime}_{0} = 1.0$$.
4. If $\left( {P_{0}^{j} } \right)^{\prime } > 0$, and $\left( {P_{0}^{j} } \right)^{\prime \prime } < 0$, then $P_{0}^{j} = \left( {P_{0}^{j} } \right)^{\prime }$, $f_{{{\text{h1}}}}^{j} = \left( {f_{{{\text{h1}}}}^{j} } \right)_{s}$, $\eta_{0} = \eta^{\prime}_{0} = 0.5$.
5. If $\left( {P_{0}^{j} } \right)^{\prime } < 0$, and $\left( {P_{0}^{j} } \right)^{\prime \prime } > 0$, then $P_{0}^{j} = \left( {P_{0}^{j} } \right)^{\prime \prime }$, $f_{{{\text{h1}}}}^{j} = \left( {f_{{{\text{h1}}}}^{j} } \right)_{t}$, $\eta_{0} = \eta^{\prime\prime}_{0} = 1.0$.
6. If $\left( {P_{0}^{j} } \right)^{\prime } < 0$, and $\left( {P_{0}^{j} } \right)^{\prime \prime } < 0$, then $P_{0}^{j} = 0$, $f_{{{\text{h1}}}}^{j} = \min \left( {\left( {f_{{{\text{h1}}}}^{j} } \right)_{s} ,\left( {f_{{{\text{h1}}}}^{j} } \right)_{t} } \right)$, $\eta_{0} = 0$.

## Calculation of overall stability for the anti-inclined slope

Under the condition that the certain inclination of a slope failure surface is $\omega^{j}$, the residual downward thrust of each rock stratum can be calculated according to formulas (1)–(22). At the same time, the safety factor $f_{{{\text{s}}i}}^{j}$ or $f_{{{\text{h}}i}}^{j}$ of each rock stratum can be calculated, $\left( {1 \le i \le n} \right)$. In other words, when the failure surface is determined, the safety factor of shearing and sliding failure or bending and toppling failure of each rock stratum can be calculated, as well as the mutual thrust of each rock stratum. The downward thrust of the last rock stratum (rock stratum 1) at the foot of the slope is the residual sliding thrust of the slope. According to the stability safety factor of each rock stratum $f_{{{\text{s}}i}}^{j}$ or $f_{{{\text{h}}i}}^{j}$, the stability safety factor $f^{j}$ of the slope can be determined.

23 $$f^{j} = \min \left[ {f_{{{\text{s1}}}}^{j} {\text{ or }}f_{{{\text{h1}}}}^{j} , \cdot \cdot \cdot \cdot \cdot \cdot ,f_{{{\text{s}}i}}^{j} {\text{ or }}f_{{{\text{h}}i}}^{j} , \cdot \cdot \cdot \cdot \cdot \cdot ,f_{{{\text{s}}n}}^{j} {\text{ or }}f_{{{\text{h}}n}}^{j} } \right]$$

When $\omega^{j}$ is given different values, the stability safety factor $f^{j}$ can be obtained by reusing formulas (1)–(22), corresponding to every $\omega^{j}$. According to the actual situation, the number of $\omega^{j}$ is $m$, $\omega^{j}$ can be taken within the range of $[90^\circ - \rho ,\phi ]$, that is $j \in [1,m]$[1m], the sizes of $f^{j}$ must be unequal. The $\omega^{j}$ corresponding to the smallest $f^{j}$ is the inclination of the overall failure surface (or the potentially most dangerous failure surface) most likely to be formed in the slope. At this time, the overall stability safety factor is recorded as $F$,

24 $$F = \min \left[ {f^{1} ,f^{2} ,f^{3} , \cdot \cdot \cdot \cdot \cdot \cdot ,f^{{{\text{m}} - 2}} ,f^{{{\text{m}} - 1}} ,f^{{\text{m}}} } \right]$$

**Figure 9:**
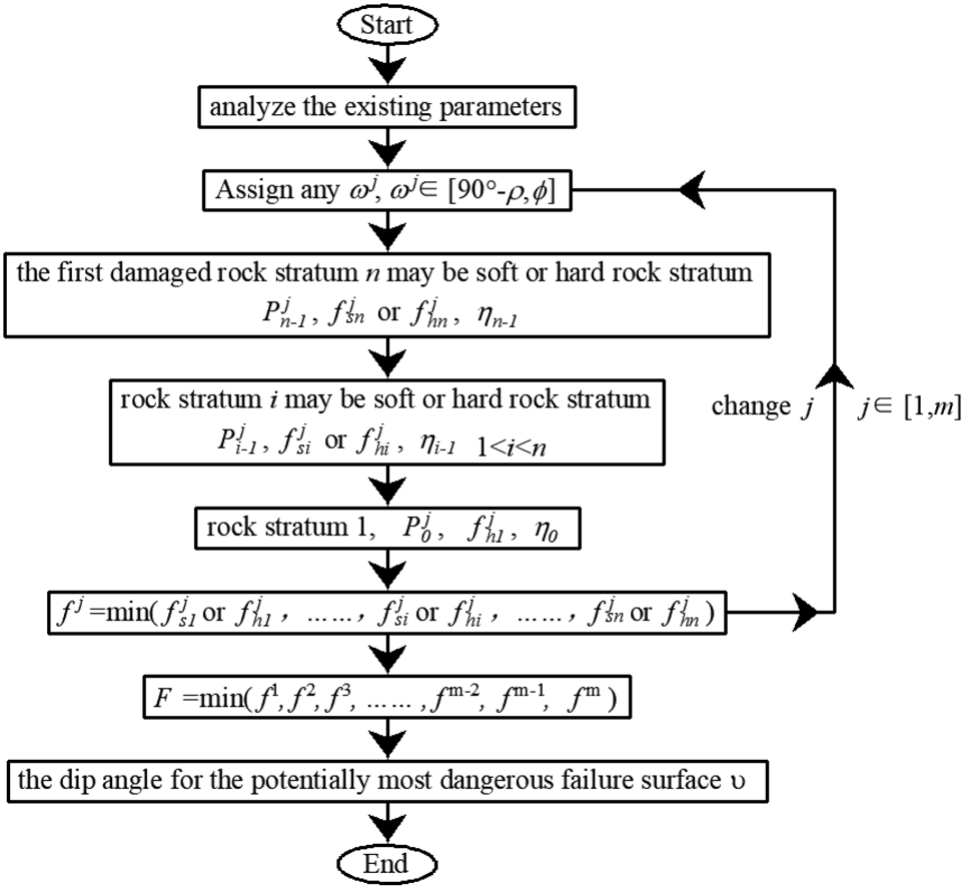
The flow of the calculation for the dip angle of the potentially most dangerous failure surface.

The flow of the calculation is shown in Fig. 9 below.

## Case analysis and verification

Based on the landslide of Gongjiafang-Dulong section Three Gorges Reservoir area, the centrifugal model test and discrete element numerical simulation of soft–hard-interbedded anti-inclined rock slope are completed in the literature^17^. The forming process of deformation and toppling failure mechanism for this kind of slope are studied. However, the theoretical analytical solution of the stability safety factor for this soft–hard-interbedded anti-inclined rock slope is not given. Based on the case in the literature, this study analyzes the potential dangerous failure surface of the slope by using the computational formula of overall stability safety factor deduced above, and it is compared with the physical model test and numerical simulation results obtained in the literature^17^. Specific parameters include:

① The dip angle of rock stratum is equal to the slope inclination, $\rho { = }\phi { = }60^\circ$.
② The thickness of hard rock stratum is equal to that of soft rock stratum, $t_{{\text{h}}} { = }t_{{\text{s}}} { = }1.5{\text{m}}$.
③ The number of soft rock strata is 11, the number of hard rock strata is the same.
④ The gravity of soft rock stratum $\gamma_{{\text{s}}} = 22.5{\text{kN}} \cdot {\text{m}}^{ - 3}$, and the gravity of hard rock stratum $\gamma_{{\text{h}}} = 28.5{\text{kN}} \cdot {\text{m}}^{ - 3}$.
⑤ The cohesion on the cross section of soft and hard rock strata are $c_{{\text{s}}} = 1.4{\text{MPa}}$ and $c_{{\text{h}}} = 3.4{\text{MPa}}$, respectively.
⑥ The internal friction angle on the cross section of soft and hard rock strata are $\varphi_{{\text{s}}} = 15^\circ$ and $\varphi_{{\text{h}}} = 23^\circ$, respectively.
⑦ The tensile strength on the cross section of soft and hard rock strata are $\sigma_{{\text{s}}} = 1.1{\text{MPa}}$ and $\sigma_{{\text{h}}} = 2.5{\text{MPa}}$, respectively.
⑧ The cohesion and internal friction angle between soft rock stratum and hard rock stratum are $c_{{{\text{hs}}}} = 0.015{\text{MPa}}$ and $\varphi_{{{\text{hs}}}} = 12^\circ$, respectively.

**Figure 10:**
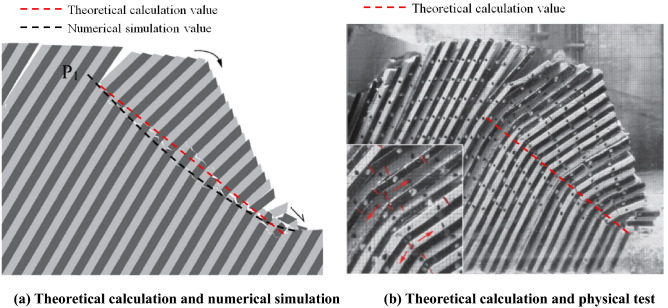
The diagram for comparison and analysis of computational results by different methods.

According to the theoretical formula provided in this study, the potential dangerous failure surface of the slope is taken as a plane nearsightedly, the dip angle for the potential dangerous failure surface of the slope is $37.5^\circ$. The overall stability safety factor is $F = 0.98$. In the two-dimensional calculation and analysis, the overall failure line of rock stratum obtained by theoretical calculation is compared with the physical model test and numerical calculation results in the literature, as shown in Fig. 10. The failure surface of the slope obtained from the theoretical computational formula proposed in this study is basically consistent with the failure surface of the slope obtained from physical model test and numerical calculation.

The correctness of the theoretical formula proposed in this study is proved by the above analysis. More importantly, the analytical solution in mathematics is given in this study. In engineering application, it is relatively simple and efficient. It does not need to spend a lot of time on building physical model tests and establishing numerical analysis models.

## Analysis of sensitive influencing factors

**Figure 11:**
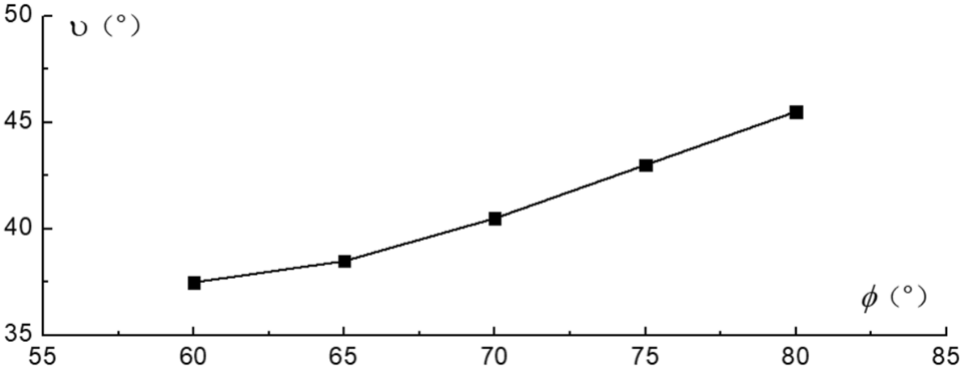
Curves for dip angles of failure surface vs dip angles of slope surface.

**Figure 12:**
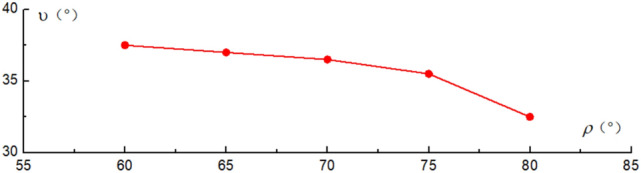
Curves for dip angles of failure surface vs dip angles of rock strata.

Relationship between dip angles of failure surface with dip angles of slope surface are shown in Fig. 11. Relationship between dip angles of failure surface with dip angles of rock strata are shown in Fig. 12. The depth of toppling deformation and failure is mainly controlled by the dip angle of rock strata. The greater the dip angle of rock strata, the smaller the dip angle of failure surface, and the greater the depth of toppling deformation in the slope. As the dip angles of slope surface increases, so does the dip angle of failure surface. According to the Figs. 11 and 12, such a conclusion can be drawn: for the anti-inclined rock slope, the dip angle of slope surface has a greater impact on the slope stability than the dip angle of rock strata. This conclusion is also consistent with the conclusion in the literature.

**Figure 13:**
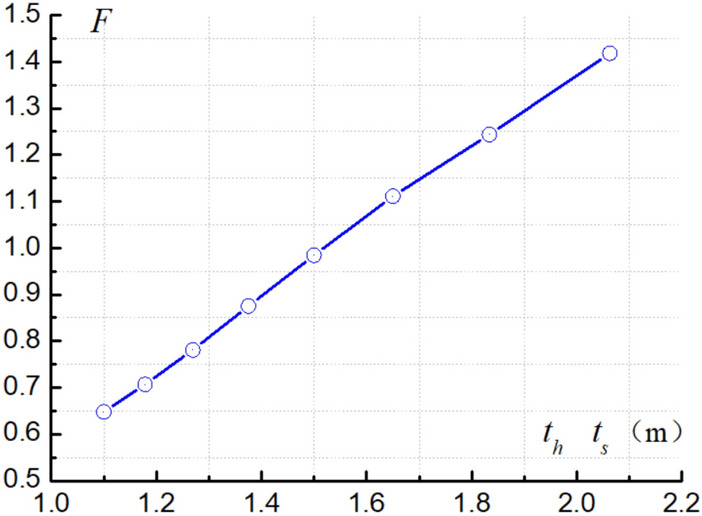
Curves for the thickness of rock strata vs the safety factor of the slope.

In order to check the effect of changing the thickness of the rock layers on the safety factor of the slope, assuming that the height of the slope remains constant, when the thickness of rock strata changes constantly, the overall stability safety factor $F$ of the slope is calculated, and the relationship curve between the thickness of rock strata and the safety factor of the slope is obtained, as shown in the Fig. 13. The thicker the hard or soft rock strata are, the greater the overall safety factor of the slope is.

## Conclusion and discussion

In this study, a theoretical method for the stability analysis of soft–hard-interbedded anti-inclined rock slope is presented. The geomechanical model of the anti inclined slope with interbeddings of soft and hard rocks is established. Then, based on the limit equilibrium theory, the mode of failure and the mode of force transmission for each anti-inclined rock stratum are discussed. Thirdly, the theoretical method for calculating the overall stability of the anti-inclined slope with interbeddings of soft and hard rocks is proposed, the theoretical solving method proposed in this study can also solve the location of the potentially most dangerous failure surface of soft–hard-interbedded anti-inclined rock slope. Finally, the experimental research results of other scholars are compared with the theoretical solving results proposed in this study. This conclusion is also consistent with the conclusion in the literature. According to the theoretical solving method proposed in this study, the theoretical calculation result is in good agreement with the results of physical experiments and numerical calculations. Compared with the physical test method and numerical method involved in the existing research results, the theoretical computational method proposed in this study is simpler and more convenient.

Dip angles of slope surface $\phi$, dip angles of rock strata $\rho$ and the thickness of the rock layers are the sensitive influencing factors for the overall stability of the soft–hard-interbedded anti-inclined slope. The dip angle of slope surface has a greater impact on the slope stability than the dip angle of rock strata. The thicker the rock stratum is, the greater the safety factor is.

**Figure 14:**
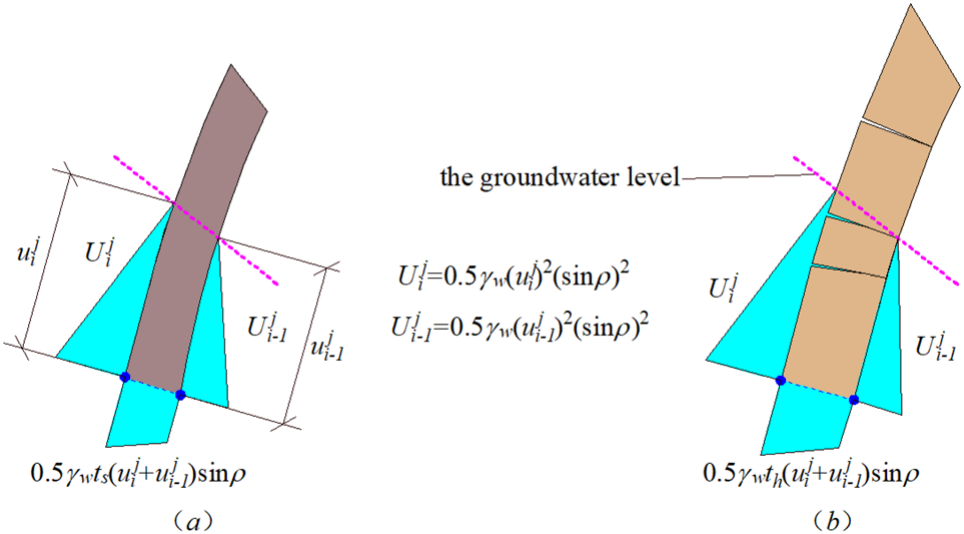
Stress analysis diagram of rock stratum under water pressure.

For the rock slope below the groundwater level, the effect of water should be taken into account in the theoretical computational method proposed in this study. The influence of water is analyzed as shown in the Fig. 14, the gravity of water is expressed as $\gamma_{w}$. When the slope is below the groundwater level, the pressure of water can be added to the theoretical computational method proposed in this study.

Though the proposed theoretical method can determine the location of the potentially most dangerous failure surface of soft–hard-interbedded anti-inclined rock slope, the overall failure surface is assumed to be plane. This is not completely consistent with the actual situation. Therefore, a more accurate theoretical method needs to be further studied.

## Data Availability

All data generated during this study are included in this published article.
